# Increased long-term health risks attributable to select volatile organic compounds in residential indoor air in southeast Louisiana

**DOI:** 10.1038/s41598-020-78756-7

**Published:** 2020-12-10

**Authors:** Jeffrey K. Wickliffe, Thomas H. Stock, Jessi L. Howard, Ericka Frahm, Bridget R. Simon-Friedt, Krista Montgomery, Mark J. Wilson, Maureen Y. Lichtveld, Emily Harville

**Affiliations:** 1 Department of Environmental Health Sciences, School of Public Health and Tropical Medicine, Tulane University, 1440 Canal Street, Suite 2100 #8360, New Orleans, LA 70112 USA; 2 Department of Epidemiology, Human Genetics and Environmental Sciences, School of Public Health, The University of Texas Health Science Center At Houston, 1200 Pressler Street, Houston, TX 77030 USA; 3 Department of Epidemiology, School of Public Health and Tropical Medicine, Tulane University, 1440 Canal Street, New Orleans, LA 70112 USA

**Keywords:** Risk factors, Cancer

## Abstract

Volatile organic compounds (VOCs) represent a broad class of chemicals, many of which can be found in indoor air including residential indoor air. VOCs derive from a variety of sources including cleaning products, cooking practices, fragrances and fresheners, hobbies and at-home work behaviors. This study examined residential indoor air in homes (n = 99) in southeast Louisiana using passive organic vapor monitors and gas chromatography/mass spectrometry to determine if select VOCs were present, at what concentrations, and if those posed any potential long-term health risks. Twenty-nine VOCs were targeted in cross-sectional analyses using a 48-h sampling period. Twelve VOCs were detected in most of the homes sampled including xylenes, pinenes, benzene, toluene, ethylbenzene, hexane, pentane, chloroform, and carbon tetrachloride. Concentrations of alkanes and BTEX compounds were highly correlated (Spearman’s r > 0.63, *p* < 0.0001). Using health risk measures (i.e. reference concentrations [RfCs] and inhalation unit risks [IURs]) available from the USEPA non-cancer risk assessments and cancer risk assessments were developed for some of these VOCs. Alkanes and BTEX compounds likely come from the same indoor source(s). Using existing health standards published by the USEPA, no unacceptable non-cancer risks were evident except under extremely high concentrations. Lifetime cancer risks, on the other hand, may well be considered unacceptable for chloroform and benzene (upper IUR) and for the combination of chloroform, benzene, and carbon tetrachloride. These exceeded a 1 in 10,000 cancer risk threshold in 35–50% of our simulations. Further study of residential indoor air in low-income women’s homes in this area is needed. Including a larger number of VOCs may reveal yet more potential health risks.

## Background

According to time budget research in the US and in Europe, people spend over 90% of their time indoors and around 70% of that indoor time in the home^1^. Therefore, indoor air quality is as, or more, important than outdoor air quality when it comes to human exposures to many air pollutants and the effects on public health. While this indoor time includes a considerable amount of time spent in the workplace, much of this time involves time spent in the home. In either situation this is important as the concentrations of some air pollutants can be much higher (2 to 5 times) indoors than they are outdoors^2^. While indoor air pollutants have been relatively understudied compared to outdoor air pollutants, there is a growing body of research demonstrating the importance and complexity of indoor air pollution^3–18^. While there is obvious exchange between indoor and outdoor air, indoor air pollution and chemistry are quite different in comparison to outdoor air pollution and chemistry^3,19^. A number of recent studies focused on indoor air chemistry that are part of the program Indoor Chem (https://indoorchem.org/) are now documenting the complex and dynamic nature of indoor air pollutants, their sources, and the variety of indoor chemical reactions that produce them^3,19–24^. Food preparation and cooking, cleaning products and practices, hobbies, arts and crafts, furnishings, building materials, work activities, behavioral practices (e.g. smoking, vaping, burning candles or incense), and personal care product use all drive the production of a number of air pollutants many of which are volatile organic compounds or VOCs^3,13,14,19–21,23,25–28^. In addition, a number of these compounds take part in secondary or downstream reactions producing additional pollutants, some of which are themselves highly reactive^3,19,21,22,24–26,29^. Thus, indoor air pollution and related human exposures are complex and dynamic.

Characterization of exposure to air pollutants and related health risk assessments and health policies in most of the world have focused mostly on outdoor pollution. This is likely because of the nature of regulating air pollutants as well as the relative ease with which outdoor sources, either point or mobile, can be regulated. Developing broad human and environmental health regulations for indoor air pollution would practically be much more difficult especially in residential, personal environments. In part, this is because of the person-specific and perhaps voluntary nature of exposure to indoor air pollution, especially residential exposures. However, a fuller investigation of indoor air pollutants, their sources, and possible health consequences is essential to further educate the public, inform safer product development and use, and improve environmental and human health. A series of studies examining exposure to air pollution have demonstrated the importance of indoor air in driving the most relevant exposures. These studies, components of the research program entitled Relationships of Indoor, Outdoor, and Personal Air (RIOPA) have shown (through measuring air pollutants outside homes, inside homes, and direct personal monitoring) that indoor and personal measurements are highly correlated^30–32^. Indoor air or personal measurements are not well correlated with outdoor air measurements^30–32^. Furthermore, measurements provided by stationary ambient-air monitoring stations show virtually no relationship with indoor air and personal measurements^30–32^.

The current lack of knowledge regarding indoor air pollution and human exposures likely explains, at least in part, the fact that studies examining health outcomes related to outdoor air pollutants are often only successful in explaining a small proportion of the actual health effects. For example, studies of exposures to outdoor air particulate matter, usually fine particulate matter (e.g. PM_2.5_), do find significant and biologically plausible associations with adverse health outcomes such as respiratory and cardiovascular disease, but the PM_2.5_ exposures explain only a small proportion of the apparent effects on health^33^.

To date, relatively few studies have gone beyond measuring indoor air pollution including specific compounds and chemicals and attempted to assess possible residential health risks^13,34,35^. The study by Hun and colleagues^34^ examining deterministic individual VOC cancer risks and combined or cumulative cancer risks from 12 VOCs did find that lifetime risks in the studied metropolitan areas were excessive and were especially so among minority Hispanic Whites. Studies in China found that lifetime cancer risks were excessive for many of the same indoor VOCs examined in studies in the US (e.g. RIOPA) based on probabilistic simulations^36,37^. The study described here aimed to determine the levels of specific VOCs in the indoor air of homes of low-income women in southeast Louisiana using a cross-sectional design. By way of additional background, the immediate rationale for this project was the Deepwater Horizon oil spill in 2010, and the concern among communities in southeast Louisiana about impacts on their air quality. This included possible increases in oil spill chemicals in residential outdoor and indoor air. This project was an outgrowth of several community-based discussions between the researchers and affected communities in Orleans, LaFourche, and Terrebonne parishes and was developed to assess residential indoor air quality in low-income homes within these communities on a small scale for the first time. To date, no research has been conducted on indoor air pollution in residences in southeast Louisiana or possible non-cancer and cancer risks associated with VOCs in homes in this region. As such, another aim of this study was to assess the acute and chronic health risks (i.e. non-cancer and cancer) that may be attributed to such exposures. To accomplish this aim, a probabilistic framework was used to assess individual VOC risks as well as combined or cumulative risks for multiple VOCs where possible. This was carried out following specific guidance and recommendations currently in use by the United States Environmental Protection Agency^38^.

## Methods

### Study subjects

Human subjects recruited for this study were selected from a larger cohort study of women of reproductive age (18–45 years old) examining the effects of the Deepwater Horizon oil spill on birth outcomes, seafood consumption and health risks, and measures of stress and anxiety^39–43^. Most of the women in this study were eligible for the Women, Infants, and Children program (WIC) operated by the U.S. Department of Agriculture and were predominantly recruited from WIC-serving clinics in southeast Louisiana. Up to 100 women were approved for enrollment in this study from 2013 to 2015. They were asked to provide basic demographic information including self-reported race/ethnicity, age, household income, and education. Racial and ethnic categories were those defined by the National Institutes of Health in NOT-OD-15-089, and these are American Indian or Alaska Native, Asian, Black or African American, Hispanic or Latino, Native Hawaiian or Other Pacific Islander, or White. Women were also asked to report if they smoked tobacco or not. Answers to this question were by self-report and were dichotomized as a Yes or No response. This study was approved by the Tulane University Biomedical Institutional Review Board under protocol 262,504. All methods were performed in accordance with the relevant guidelines and regulations. All participants provided written, informed consent prior to enrollment.

### Air sampling

Air was sampled at participants’ residences using a paired indoor-outdoor approach. Passive diffusion monitors (3M Organic Vapor Monitors, OVM3500) were deployed for approximately 48 h inside and outside of each residence. Samplers were placed in proximity to primary living areas and not bedrooms and in areas directly out of strong air flow. They were placed approximately 1.5–2.0 m off of the ground, at least 0.5 m from any wall to allow for adequate air circulation, and away from any known VOC sources essentially following the approach used by Weisel et al.^30^. A field and laboratory blank was included during each sampling session to account for background or any contamination^44^. Air sampling was carried as part of a larger project examining maternal mental health and seafood quality and safety following the Deepwater Horizon oil spill in 2010. These samplers have been successfully deployed in previous studies of VOCs in indoor, outdoor, and personal air^30,31,44^. Air sampling was carried out in 6 separate field deployment sessions. Data reported herein is restricted to the indoor air samples and does not include an in-depth reporting or analysis of the outdoor air samples. Residences varied from single-family homes, townhomes, duplex homes, apartments, manufactured homes, mobile homes, and trailers. Because of the small sample size, we did not stratify results based on residence type. Twenty-nine VOCs were targeted and are presented in Table 1. They are considered important compounds that can affect human health, and many of them have a strong enough evidence base from which to estimate human health inhalation risks. This scientific evidence base has been used to develop the appropriate reference concentrations (RfCs) for estimating non-cancer risks and inhalation unit risks (IURs) for estimating cancer risks. These have been peer-reviewed and published by the USEPA in the Integrated Risk Information System database. General sources, links to chemical descriptions, product sources, and toxicological information are also provided in Table 1. PubChem (https://pubchem.ncbi.nlm.nih.gov/) was used to provide a source for specific information on each VOC, sources and products that VOCs are found in, and possible toxicological information regarding VOCs respectively where possible. This site was developed and maintained by the U.S. National Library of Medicine of the National Institutes of Health.

**Table 1:** Targeted volatile organic compounds (VOCs) in residential air sampling in southeast Louisiana.

VOC	General sources including links for detailed information
m,p-Xylene	Cigarettes, degreasers, solvents, spray lubricants PubChem
o-Xylene	Cigarettes, gasoline, paint, paint thinner PubChem
alpha-Pinene	Food flavoring, pine scented cleaners, odor masking products PubChem
Toluene	Cigarettes, gasoline, solvents, adhesives, paint, aerosols, pest control PubChem
d-Limonene	Personal care products, fragrance, perfume, solvent, insecticide, cleaners, food flavoring PubChem
Ethylbenzene	Cigarettes, paint, sealants, automotive products, insecticide PubChem
Chloroform	Byproduct of chlorination of water, solvent, refrigerant PubChem
Hexane	Adhesives, sealants, paint, craft supplies PubChem
Pentane	Adhesives, lubricants, personal care products, fuels, plastics, home building and construction materials PubChem
Carbon tetrachloride	Cigarettes, solvent, degreaser, adhesive remover PubChem
beta-Pinene	Food flavoring, pine scented cleaners, odor masking products, laundry and dishwashing products PubChem
Benzene	Cigarettes, gasoline, solvent, adhesive remover, motor oil PubChem
1,2,3-Trimethylbenzene	Fuel injector cleaner, fuel and additives, solvent PubChem
p-Dichlorobenzene	Odor masking products, moth balls, resins PubChem
1-Ethyl-2-methylbenzene	Cigarettes, solvent, gasoline, paint, adhesives, laundry detergent, odor masking products PubChem
Nonane	Paint, coatings, solvent PubChem
Styrene	Cigarettes, auto exhaust, rubber, plastic, disposable containers PubChem
Decane	Cigarettes, solvent, fuel PubChem
1,2,4-Trimethylbenzene	Fuel, additives, solvent, paint, coatings, adhesive, herbicide PubChem
Methylene chloride	Solvent, degreaser, pesticide, paint remover, cleaning agent PubChem
Methylethylketone	Cigarettes, solvent, paint, coating, glue, printing ink, photographic products, personal care products, building materials, water treatment, fabric PubChem
1,3,5-Trimethylbenzene	Paint, paint thinner, solvent, fuel additive, coatings PubChem
Methylcyclopentane	Solvent, component of the naphthene fraction of petroleum PubChem
Tetrachloroethylene	Auto care products, household cleaners, lubricants, solvent PubChem
Trichloroethylene	Degreaser, adhesives, sealants, paint, coatings PubChem
Naphthalene	Cigarettes, moth balls, deodorizers, burning wood or fuels PubChem
Isoprene	Cigarettes, plastic, rubber, building materials PubChem
Dimethylpentane	Anti-knocking fuel additive PubChem
Methyl tert-butyl ether	Anti-knocking fuel additive, contaminated groundwater PubChem

Information including general source(s) are provided as well as products that VOCs are found in and toxicological information regarding each VOC where available. Items without active hyperlinks do not have information available at this time (accessed 19-May-2020).

### Chemical analysis

Every several weeks, all stored OVM field samples, collocated/duplicate samples and field blanks were shipped overnight in hard plastic coolers with blue ice packs to the UTSPH Environmental Analytical Laboratory. Collocated/duplicate samples are quality control samples placed side-by-side with field samples in order to be able to assess the overall precision of the sampling/analytical procedure. Two or three sampling locations per session were randomly selected for this. For example, in the first field sampling session 3 duplicate samplers were deployed in 3 homes selected at random. Samples were stored in a dedicated refrigerator until analysis, which was typically performed within 4–5 days after receipt. Extraction and analytical procedures have been described in detail previously^45^. Gas chromatography/mass spectrometry (GC/MS) analysis was performed using a HP 6890 Series GC with a 5975B MSD and EnviroQuant software. The column employed was a Restek (RTX-624, 60 m 0.25 mm ID with 1.4 um thickness column (Restek Corp., Bellefonte, PA). Samples were analyzed in 11 analytical batches. For each batch, an 8-point calibration curve was run initially, and then, after every 20 samples, a duplicate analysis of the 20th sample was run, followed by a 1.0 µg/mL standard (calibration check) and a solvent wash. Mean blank values for each batch were subtracted from all sample masses determined in that run.

Mass method detection limits (MDLs) for most compounds were determined for each batch of samples from the combined results of the analyses of field and laboratory blanks. For compounds not present as background contaminants, the mass MDL was determined from the variation of multiple analyses of a low analytical standard (0.1 µg/mL). Details regarding the determination of MDLs are provided in Chung et al.^45^. Sample-specific concentration MDLs were then calculated from the mass MDLs using the air sampling durations^44^.

### Data and statistical analysis

Descriptive statistics for all VOCs were calculated in Prism ver. 8.1.0 (GraphPad, San Diego, CA). Censored values or samples with non-detects (i.e. below the method detection limit or MDL) are not uncommon for these compounds. To screen VOCs for further analysis including health risks where a large proportion of censored values render such results largely artificial, data were subjected to distributional testing using the Kolmogorov–Smirnov (K–S) test (Prism ver. 8.1.0). In some cases, the chemical analysis yielded an actual value, but this value was below the actual MDL. These values were used as is. In other cases, the chemical analysis resulted in a non-detect, and those samples were assigned a value of 0 µg/m^3^. For this situation, a value corresponding to the $MDL\div \surd 2$ was used in place of 0 for statistical analysis purposes. The raw distributions (unadjusted) were then compared to the MDL-adjusted distributions using the K–S test, and where distributions were significantly different (*p* < 0.01), such VOCs were not included in further analysis. This was done to conservatively and objectively include only those VOCs with the most robust, complete data. Correlation analyses using the Spearman’s non-parametric rank coefficient were carried out among VOCs passing the K–S test restricting meaningful correlations to r ≥ 0.50 at *p* < 0.01. Benzene was used a proxy air pollutant produced by smoking tobacco indoors for evaluating the correlation between self-reported smoking status and indoor air VOCs produced from combustion of tobacco. Simple logistic regression was also used to examine the relationship between self-reported smoking status and indoor air benzene concentrations.

### Risk analysis

VOCs passing the K–S test were then included in probabilistic risk analyses using the risk analysis add-in for Microsoft Excel @Risk ver. 7.6.0 (Palisade Corporation, Ithaca, NY). The USEPA’s Integrated Risk Information System was also used for these analyses. VOCs with either a reference concentration (RfC) and/or an inhalation unit risk (IUR) were then assessed for either non-cancer and/or lifetime cancer risks. To carry out the probabilistic risk analyses, distributions of unadjusted VOCs were modeled using default parameters of the Batch Fit option in @Risk. The Akaike Information Criterion was used to select the best distributional fit along with the distributional parameters for each VOC^46^. These distributions were then used to carry out non-cancer and cancer risk simulations using 100,000 iterations with a Latin Hypercube sampling type and a Mersenne Twister random number generator^42,47^. Non-cancer risks were assessed using the hazard quotient (HQ) approach and the following equation, $HQ=[VOC] \div  RfC.$ This generates a unitless ratio and risks are considered excessive if the HQ > 1. Cancer risks (CR) were assessed using the following equation, $CR=[VOC] \times IUR.$ This generates a unitless probability where it is widely accepted that if the CR ≥ 1 × 10^–4^ the cancer risk is considered excessive. The USEPA often uses a benchmark or threshold CR of 1 × 10^–6^^34^. Prior to risk modeling, a minimum truncation limit of 0 µg/m^3^ was set for each VOC distribution to prevent negative values of concentrations from being selected during simulations. Hazard quotients for VOCs affecting the same organ system (e.g. liver, kidney, or hematological) were summed to assess combined non-cancer risks. We took a more conservative approach with our cancer risk estimates and assumed a lifetime of exposure beginning at birth. Therefore, we applied age-dependent adjustment factors that account for increased susceptibility to adulthood cancers resulting from pediatric and adolescent exposure to carcinogens using the USEPA’s methods^48^. We used a life expectancy of 81.1 years consistent with the U.S. Centers for Disease Control and Prevention’s (USCDC) estimate for women in the US^49^. Cancer risk probabilities for VOCs with available IURs were summed to assess combined cancer risks without regards to cancer type or specificity as is currently and conservatively recommended by the USEPA^38^.

### Ethics approval and consent to participate

This study was approved by the Tulane University Biomedical Institutional Review Board and the Human Research Protection Office at Tulane University under study identification number 262504. All research participants provided written informed consent prior to study enrollment.

## Results

### Study subjects

A total of 99 women provided informed consent and participated in this study. Seventy-seven women provided basic demographic data (Table 2). Women participating in this study were typically Black, non-Hispanic, approximately 30 years of age with an annual household income reported at < $15,000 and a high-school education or less. Seventy-two women chose to answer the question regarding smoking status (Table 2). Approximately 29% of the women in this study reported smoking tobacco which is higher than the USCDC’s Behavioral Risk Factor Surveillance System’s estimate of 19.3% (95% CI 17.3–21.3) in 2015 (BRFSS; accessed on April 10, 2020). The BRFSS estimate is for adult women in the state of Louisiana and is an on-going survey study conducted by the USCDC^50^. This difference may be due to demographic differences among this group of women and women across the state of Louisiana or some other unknown factor.

**Table 2:** Demographic characteristics of research participants in this study.

Race/ethnicity	N (%)
Black, non-Hispanic	48 (63)
Black, Hispanic	1 (1)
White, non-Hispanic	18 (24)
White, Hispanic	1 (1)
Hispanic	3 (4)
Asian	2 (3)
Other	3 (4)
Didn’t report	1 (1)

Age	Years
Median	30
25th%-ile	25
75th%-ile	33
Minimum	19
Maximum	45

Categorized annual income	N (%)
< $15,000	38 (54)
$15,000–$35,000	22 (31)
> $35,000	11 (15)
Didn’t report	6 (8)

Categorized education	N (%)
High school or less	34 (47)
Some college	30 (41)
College or more	9 (12)
Didn’t report	3 (4)

Smoking status	N (%)
Yes	21 (29)
No	51 (71)

### VOCs in indoor air

As mentioned above, only the data for indoor air samples is the subject of this report. Briefly though, indoor air levels of these 29 VOCs exceeded outdoor levels, when detected, at all residences examined and indoor and outdoor levels were weakly or modestly correlated (e.g. d-limonene, Spearman r = 0.29, 95% CI 0.08–0.48, *p* < 0.01; benzene, r = 0.60, 95% CI 0.44–0.73, *p* < 0.001). Outdoor air concentrations were much lower than indoor air concentrations and did not contribute appreciably to estimated health risks. Descriptive statistics for indoor air VOCs are presented in Table 3.

**Table 3:** Descriptive statistics for indoor air VOC data and results of K–S distributional testing.

VOC	Concentration (µg/m^3^)	Censored data (percentage of total samples) (%)	MDLs (µg/m^3^)	K–S test
m,p-Xylene	2.09 (0.63–35.36)	0	0.24 (0.06–0.58)	D = 0.01, *p* > 0.999
o-Xylene	0.81 (0.32–11.82)	0	0.25 (0.08–0.51)	D = 0.03, *p* > 0.999
alpha-Pinene	4.73 (0.90–44.33)	1	0.40 (0.12–0.80)	D = 0.01, *p* > 0.999
Toluene	4.91 (0.84–66.22)	2	0.41 (0.14–12.21)	D = 0.08, *p* < 0.95
d-Limonene	17.90 (1.79–121.20)	2	0.49 (0.13–3.23)	D = 0.02, *p* > 0.999
Ethylbenzene	0.74 (0.29–8.65)	3	0.15 (0.05–0.68)	D = 0.04, *p* > 0.999
Chloroform	1.89 (0.01–10.84)	4	0.08 (0.03–0.48)	D = 0.05, *p* > 0.999
Hexane	1.08 (< MDL-20.03)	10	0.43 (0.16–2.97)	D = 0.15, *p* < 0.24
Pentane	4.11 (< MDL-55.81)	11	1.09 (0.33–2.36)	D = 0.11, *p* < 0.65
Carbon tetrachloride	0.42 (< MDL-1.91)	11	0.27 (0.09–0.51)	D = 0.12, *p* < 0.53
beta-Pinene	2.11 (< MDL-17.35)	11	0.48 (0.16–0.90)	D = 0.11, *p* < 0.65
Benzene	1.14 (0.04–13.57)	13	0.60 (0.16–2.94)	D = 0.23, *p* < 0.017
1,2,3-Trimethylbenzene	0.47 (< MDL-8.18)	37	0.32 (0.09–0.37)	D = 0.35, *p* < 0.001
p-Dichlorobenzene	0.88 (< MDL-1043)	40	0.48 (0.23–1.68)	D = 0.41, *p* < 0.001
1-Ethyl-2-methyl benzene	0.29 (< MDL-5.96)	43	0.28 (0.09–0.54)	D = 0.44, *p* < 0.001
Nonane	0.38 (< MDL-10.97)	45	0.33 (0.11–0.62)	D = 0.44, *p* < 0.001
Styrene	0.11 (< MDL-4.20)	51	0.45 (0.20–0.74)	D = 0.50, *p* < 0.001
Decane	< MDL (< MDL-14.71)	53	0.43 (0.21–2.43)	D = 0.54, *p* < 0.001
1,2,4-Trimethylbenzene	0.16 (< MDL-5.18)	54	0.36 (0.12–0.67)	D = 0.59, *p* < 0.001
Methylene chloride	0.05 (< MDL-2.54)	55	0.41 (0.03–4.01)	D = 0.48, *p* < 0.001
Methylethylketone	< MDL (< MDL-20.46)	61	1.29 (0.27–3.43)	D = 0.52, *p* < 0.001
1,3,5-Trimethylbenzene	< MDL (< MDL-2.89)	61	0.28 (0.09–0.53)	D = 0.60, *p* < 0.001
Methylcyclopentane	< MDL (< MDL-11.13)	67	0.31 (0.10–0.58)	D = 0.51, *p* < 0.001
Tetrachloroethylene	< MDL (0.00–1.24)	79	0.27 (0.09–0.49)	D = 0.77, *p* < 0.001
Trichloroethylene	< MDL (< MDL-0.53)	83	0.23 (0.08–1.79)	D = 0.84, *p* < 0.001
Naphthalene	< MDL (< MDL-1.89)	88	0.82 (0.18–1.58)	D = 0.88, *p* < 0.001
Isoprene	< MDL (< MDL-16.09)	92	5.30 (1.81–10.22)	D = 0.89, *p* < 0.001
Dimethylpentane	< MDL (< MDL-2.76)	96	3.68 (0.15–4.36)	D = 0.88, *p* < 0.001
Methyl tert-butyl ether	< MDL	100	0.57 (0.16–1.10)	D = 1.00, *p* < 0.001

Concentrations are presented as median levels (5th percentile–95th percentile). Censored data are presented as percentages of samples out of the total number of samples below the method detection limit (MDL). MDLs (µg/m^3^) are presented as median levels (minimum and maximum).

Concentrations of most of the VOCs in indoor air were below the MDLs for their respective VOC. For seventeen of the target VOCs, ≥ 40% of the measured indoor concentrations were below the MDL. This is noteworthy as MDLs for these VOCs were in the very low µg/m^3^ or lower.

Twelve of the VOCs passed the K–S test and were included in additional analyses. These VOCs all had fewer than 15% non-detects or samples below the MDL out of the total number of samples. These are presented in Table 4 along with Integrated Risk Information System values corresponding to the RfC and/or the IUR where available.

**Table 4:** Indoor air VOCs passing the K–S test that were used for probabilistic risk analysis.

VOC	Reference concentration (µg/m^3^)	Human system affected	Inhalation unit risk (µg/m^3^)^−1^
m,p-Xylene	100 (last updated 2003)^b^	Nervous	N/A^a^
o-Xylene	100 (last updated 2003)	Nervous	N/A
alpha-Pinene	N/A^a^		N/A
Toluene	5000 (last updated 2005)	Nervous	N/A
d-Limonene	N/A		N/A
Ethylbenzene	1000 (last updated 1987)	Developmental	N/A
Chloroform	N/A		2.0E−5 (last updated 2001)
Hexane	700 (last updated 2005)	Nervous	N/A
Pentane	N/A		N/A
Carbon tetrachloride	100 (last updated 2010)	Hepatic	6.0E−6 (last updated 2010)
beta-Pinene	N/A		N/A
Benzene	30 (last updated 2003)	Immune	2.2E−6 to 7.8E−6 (last updated 2000)

Reference concentrations for non-cancer assessments and inhalation unit risks for cancer assessment are provided where available in the Integrated Risk Information System (EPA). For VOCs with an established reference concentration, the human organ or physiological system affected is included.

^a^N/A-not available in IRIS.

^b^Indicates the year in which risk information was last updated by the USEPA.

The alkanes, pentane and hexane, were highly correlated with one another and with the BTEX compounds benzene, toluene, ethylbenzene, and xylenes (Table 5). Not surprisingly, alpha-pinene and beta-pinene were highly correlated as they are derived from the same sources (Spearman’s r = 0.83, *p* < 0.001). Chloroform and d-limonene were correlated with one another (Spearman’s r = 0.54, *p* < 0.001), and d-limonene was correlated with the pinene VOCs (Spearman’s r ≥ 0.59, *p* < 0.001). Chloroform was modestly correlated with the pinene VOCs (Spearman’s r > 0.42, *p* < 0.001). Chloroform and carbon tetrachloride were weakly correlated with one another (Spearman’s r = 0.41, *p* < 0.001). Benzene concentrations and self-reported smoking status were not correlated in this study (Spearman’s r = 0.08, *p* > 0.52). Simple logistic regression also did not indicate that benzene concentration was a predictor of smoking status (β0 = −1.10, 95%CI − 1.82 to − 0.44; β1 = 0.06, 95%CI − 0.12 to 0.22; log-likelihood ratio = 0.46, *p* < 0.50).

**Table 5:** Correlations (Spearman’s r, lower left) among indoor air concentrations of pentane, hexane, benzene, toluene, ethylbenzene, and the xylenes. All correlations were significant (*p* values upper right).

	Pentane	Hexane	Benzene	Toluene	Ethylbenzene	m,p-Xylene	o-Xylene
Pentane		*p* < 0.0001	*p* < 0.0001	*p* < 0.0001	*p* < 0.0001	*p* < 0.0001	*p* < 0.0001
Hexane	0.71		*p* < 0.0001	*p* < 0.0001	*p* < 0.0001	*p* < 0.0001	*p* < 0.0001
Benzene	0.64	0.64		*p* < 0.0001	*p* < 0.0001	*p* < 0.0001	*p* < 0.0001
Toluene	0.66	0.72	0.69		*p* < 0.0001	*p* < 0.0001	*p* < 0.0001
Ethylbenzene	0.63	0.71	0.77	0.85		*p* < 0.0001	*p* < 0.0001
m,p-Xylene	0.66	0.71	0.79	0.81	0.96		*p* < 0.0001
o-Xylene	0.65	0.70	0.76	0.80	0.96	0.97	

### Risk analysis

**Figure 1:**
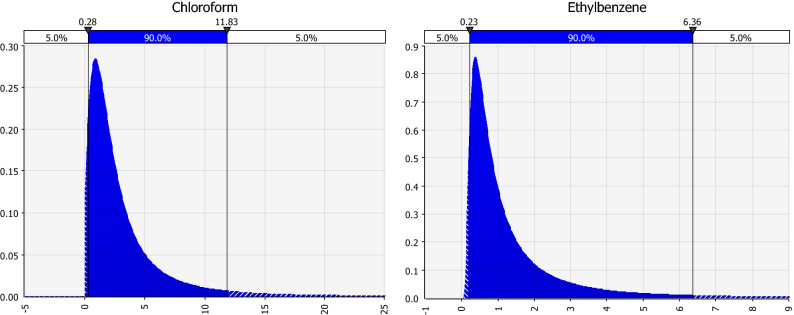
Fitted distributions of indoor air VOC data for two representative compounds, chloroform and ethylbenzene using @Risk. For both compounds, the graphs represent the respective probability density functions, the x-axis represents the airborne concentration (µg/m^3^) of the VOC, and the y-axis represents the probability density. The indoor air data for chloroform were best fit by a log-logistic distribution, and the indoor air data for ethylbenzene were best fit by a Pearson5 distribution.

Distributional batch fitting of the VOCs that were used in the risk analyses indicated that indoor air concentrations were fit by a log-logistic distribution best, except for ethylbenzene and the xylenes which were fit by a Pearson5 distribution best. Both are similar in shape to a log-normal distribution. Representative distributions are shown in Fig. 1.

Median non-cancer risks (hazard quotients) for individual VOCs were below 1 for all of the compounds with RfC values indicating no excess non-cancer risks are being experienced by this group of women. Only at the highest percentile (≥ 99%) did hazard quotients marginally exceed 1 for benzene and the combined VOCs (hexane, toluene, and mixed xylenes). We note here that some chemicals and compounds can have toxic effects that cause both non-carcinogenic and carcinogenic health outcomes. Hazard quotients (non-cancer risk assessments) for individual VOCs and summed VOCs are presented in Table 7.

**Table 6:** Non-cancer risk hazard quotients for VOCs with a corresponding reference concentration in the integrated risk information system.

VOC	Hazard quotient (mean, median)	Percentiles (1st, 25th, 75th, 99th)
Mixed Xylenes	0.10, 0.04	8E−3, 0.02, 0.08, 0.90
Toluene	0.003, 0.001	3E−5, 5E−4, 2E−3, 0.03
Ethylbenzene	0.002, 0.001	2E−4, 5E−4, 2E−3, 0.02
Hexane	0.018, 0.002	2E−5, 6E−4, 5E−3, 0.20
Carbon tetrachloride	0.005, 0.005	4E−4, 3E−3, 7E−3, 0.02
Benzene	0.145, 0.035	7E−4, 0.015, 0.085, 1.37
Combined^a^	0.13, 0.05	0.01, 0.03, 0.09, 1.02

Hazard quotients ≥ 1 indicate excess risk.

Cancer risks generally exceeded a threshold of 1 in 1,000,000 for chloroform, benzene, and carbon tetrachloride (Table 6). The median cancer risk exceeded a threshold of 1 in 100,000 for chloroform and benzene using the upper IUR. Median summed cancer risks exceeded a threshold of 1 in 100,000 using the lower IUR for benzene and were at the threshold of 1 in 10,000 using the higher IUR for benzene. Cancer risks exceeded the 1 in 10,000 threshold using either IUR for benzene at or above the 75th percentile even exceeding the 1 in 1000 threshold at or above the 99th percentile.

**Table 7:** Cancer risk probabilities for VOCs with corresponding inhalation unit risks in the integrated risk information system.

VOC	Cancer risk probability (mean, median)	Percentiles (1st, 25th, 75th, 99th)
Chloroform	1.2E−4, 6.3E−5	2.3E−6, 3.2E−5, 1.2E−4, 9.5E−4
Carbon tetrachloride	4.9E−6, 4.2E−6	3.8E−7, 2.7E−6, 6.1E−6, 1.7E−5
Benzene-Lower IUR	1.4E−5, 3.6E−6	7.6E−8, 1.5E−6, 8.6E−6, 1.4E−4
Benzene-Higher IUR	4.9E−5, 1.3E−5	2.7E−7, 5.3E−6, 3.1E−5, 4.9E−4
Combined^a^	1.4E−4, 7.8E−5	9.6E−6, 4.4E−5, 1.4E−4, 1.0E−3
Combined^b^	1.8E−4, 9.6E−5	1.4E−5, 5.6E−5, 1.7E−4, 1.3E−3

Probabilities at or greater than 1 in 10,000 (≥ 1E−4) are widely viewed as excessive and unacceptable.

^a^Using lower IUR for benzene.

^b^Using higher IUR for benzene.

## Discussion

### Summary-VOC concentrations

Many of the targeted VOCs in this study were detected at very low concentrations and in a fraction of our participants’ homes. With some exceptions, results for many of the VOCs in this study were similar to those in other areas of the US including Los Angeles, CA, Elizabeth, NJ, Detroit, MI, and Houston, TX^13,31^. These studies found indoor air concentrations of the BTEX compounds, styrene, chloroform, and 1,4-dichlorobenzene similar to those reported here. Studies in China, Europe, and Canada have generally found similar or slightly higher concentrations of some of these same compounds in residential indoor air^17,18,36,37^. A few of the VOCs were not detected in the indoor air of any homes. This suggests that if these VOCs are present in the indoor air of these homes, they are at very low concentrations down into the ng/m^3^ range or lower. Twelve (40%) of the VOCs were regularly found in most or all of the homes sampled usually at low concentrations (low µg/m^3^). These include the BTEX compounds (benzene, toluene, ethylbenzene, and xylenes), alkanes (hexane and pentane), chlorinated compounds (chloroform and carbon tetrachloride), and terpene compounds (alpha-pinene, beta-pinene, and d-limonene).

### VOC correlations and possible sources

Concentrations of the BTEX compounds were highly correlated and were also highly correlated with the two alkane compounds, pentane and hexane. This may indicate a common source or sources for these VOCs. These compounds are common components of fuels including gasoline as well as a number of solvents and glues. BTEX compounds are also common constituents of tobacco smoke. Smoking prevalence is still high in Louisiana especially among low-income residents. Interestingly, self-reported smoking status and benzene concentrations were not correlated in this study. Thus, it would appear that tobacco smoking among some participants was either done outside or at least not in proximity to the samplers during deployment. This further suggests that the source(s) of benzene and BTEX compounds is (are) not tobacco smoking among these women. As expected, alpha- and beta-pinene were highly correlated. These were also correlated with d-limonene. This indicates that scented products, such as household cleaners, containing these VOCs are the likely sources of these compounds^21^. Chloroform was highly correlated with d-limonene and weakly correlated with the pinene VOCs. Chloroform sources include chlorinated water (residential water) and the use of chlorine bleach^22,24,29^. Correlations among these compounds are probably a result of residential cleaning practices and product use. Chloroform and carbon tetrachloride were also weakly correlated. Products containing chlorine and the use of such products is the likely source of these chlorinated VOCs^24,25,29,51^.

### VOC risks

#### Non-cancer risks

Non-cancer risks were assessed for seven of the VOCs. These include the xylene isomers, m,p-xylene and o-xylene, toluene, ethylbenzene, hexane, carbon tetrachloride, and benzene. Non-cancer risks for the xylene isomers used a mixtures approach and combined them as mixed xylenes. No excess risks were observed for mixed xylenes, toluene, ethylbenzene, hexane, or carbon tetrachloride as individual VOCs.

Elevated or excess non-cancer risks for benzene were seen at the highest percentiles (> 95th percentile). This would suggest that under conditions of the highest exposures as measured in this study there may be a very small proportion of this population at elevated or excess risk. However, this would mean that the concentrations of benzene under these conditions would have to remain at such high indoor air concentrations (≥ RfC of 30 µg/m^3^) for extended periods, decades to a lifetime. Though this study cannot address that question unequivocally (see limitations below), it is highly unlikely that indoor air concentrations of benzene will remain consistently that high over many years even in a small number of residences. The maximum concentration of benzene observed in this study was 21.4 µg/m^3^ which is approaching the RfC. While none of the homes sampled had indoor air benzene concentrations at or above the RfC, 3.4% of the homes had levels that were ≥ 50% of the RfC.

Hexane, toluene, and the mixed xylenes were included in a mixtures or cumulative non-cancer risk assessment. These VOCs all affect the neurological system. As with benzene, excess non-cancer risks were observed only at the highest percentiles (> 95th percentile). This again suggests that only a very small proportion of homes would experience elevated or excess risks. Mixed xylenes drove most of the elevated risk as these compounds have the lowest RfC among those included in the cumulative assessment. The simulation results indicate that for 82.7% of the iterations where excess risks were observed, levels of the xylenes or mixed xylenes were at or above the RfC of 100 µg/m^3^. It is worth mentioning that the maximum concentrations of hexane, toluene, m,p-xylene, and o-xylene that were measured in this study were 66.6 µg/m^3^, 3855.5 µg/m^3^, 62.3 µg/m^3^, and 18.5 µg/m^3^ respectively. Combining the maximum values for the m,p-xylene and o-xylene isomers as mixed xylenes yields a concentration of 80.8 µg/m^3^. For mixed xylenes, 4.5% of the homes sampled in this study had indoor air concentrations ≥ 50% of the RfC, and one of these homes had an indoor air concentration of 80.8 µg/m^3^. The extreme observation for toluene could be considered an outlier as the next highest concentration observed was 144.5 µg/m^3^. It is likely that the maximum observed concentration of toluene was related to in-home product use such as paints during or very near the time of air sampling and as such may not necessarily reflect long-term or sustained concentrations of toluene at such a high level. It is also worth noting that this high level of toluene is still below the RfC of 5000 µg/m^3^.

One VOC which did not meet the conservative criteria for inclusion in the risk assessment process but is worth discussing is p-dichlorobenzene. Most of the homes had levels of this VOC that were extremely low with approximately 47% of those sampled having concentrations below the MDL. However, a small number (5.4%) of the homes sampled had indoor air concentrations of p-dichlorobenzene that exceeded the RfC for this compound (800 µg/m^3^) by an average of 774 µg/m^3^. Even though this particular VOC was not included in this risk analysis, some of these particular exposures may be of concern. This VOC affects the hepatic system. Sources of this VOC are somewhat more specific as this compound is a common component of certain pesticides, insect repellants, disinfectants, and bathroom products (e.g. toilet bowl deodorizers).

#### Chronic, cancer risks

Cancer risks were assessed for three of the VOCs. These include chloroform, carbon tetrachloride, and benzene. For all three individual compounds using the USEPA’s cancer risk threshold of 1E-6 (1 out of 1,000,000), cancer risks at the average or median indoor air concentrations would be considered excessive or unacceptable. If using a conservative cancer risk threshold of 1E-5, average or median cancer risks for chloroform and benzene (upper IUR) would still be considered unacceptable. This is true for benzene using the lower IUR at or above the 80th percentile and for carbon tetrachloride at or above the 95th percentile. Cancer risks for chloroform and for benzene using the higher IUR exceeded a 1E-4 risk at or above the 70th and 95th percentiles respectively. For chloroform, this suggests that a notable proportion of homes (~ 30%) have unacceptably high indoor air concentrations from a lifetime cancer risk perspective. Estimated cancer risks regarding benzene are, or would be, similar, using our approach, to those found in studies in the U.S., Europe, Canada, and China^17,18,36,37^.

As would be expected, chloroform drives most of the cancer risk when using a combined or cumulative approach because of its relatively high IUR. Cumulative cancer risks using either IUR for benzene indicate average or median risks exceed a 1E-4 and 1E-5 threshold respectively. When using the low IUR for benzene, cumulative cancer risks exceed a 1E-4 threshold effectively at the 60th percentile. When using the high IUR for benzene, cumulative cancer risks exceed a 1E-4 threshold effectively at the median. Thus, approximately 50% of the homes in this small study have cumulative cancer risks that exceed what is widely considered the least conservative risk threshold of 1E-4 (1 out of 10,000). Overall, cancer risks estimated in this study and those by Hun et al. (2009) were not dissimilar^34^.

As with many environmental health assessments and studies aiming to better understand exposures, this project has limitations. One major limitation of this study is that the design is cross-sectional and indoor air concentrations of these VOCs were measured only once. An assumption that is made is that these measured levels represent typical concentrations that would be found consistently or on average in homes that are sampled. However, longitudinal, repeated measurements of concentrations, as well as determinations of air exchange rates, are needed to better support such assumptions and understand the variability and dynamic nature of the indoor air chemical environment. A second major limitation of this study is that only a fraction of the VOCs in the indoor air chemical environment was measured. Measuring and determining concentrations of VOCs in the indoor air environment is technically challenging and, frankly, costly, but emerging research clearly documents that chemicals in indoor air are numerous and indoor air chemistry itself is complex and dynamic^3,14,19^. To more comprehensively address potential health risks, a more complete panel of VOCs will be required for such assessments. A larger study including many more homes as well as a much more extensive characterization of the residences, product usage, resident behaviors and time-activity patterns, cleaning practices, hobbies, and cooking practices would also improve risk assessments, reducing uncertainty and biases towards overestimates of risk, and identification of sources in future studies. Finally, no health data were directly measured (at least for this analysis) so a better integration of health outcomes and exposure analysis would be more informative and better address knowledge gaps in this area of public health research.

## Conclusions

Using a probabilistic framework and the USEPA’s standard methodology, non-cancer risks were largely acceptable for the VOCs that were available for a robust assessment. Cumulative non-cancer risks were also largely acceptable and not considered excessive. Excess risks were observed in simulated iterations at or above the 99th percentile, but the conditions required to achieve such risks are considered highly improbable. One VOC, p-dichlorobenzene, not included in the probabilistic risk assessment approach, is still of concern as a small number of homes sampled had indoor air concentrations that were effectively twice that of the RfC.

Using the USEPA’s cancer risk threshold of 1E-6, individual VOC cancer risks as well as cumulative cancer risks would be considered unacceptable and excessive. Even using the least conservative risk threshold of 1E-4, cancer risks for chloroform, benzene, and cumulative cancer risks may well be considered excessive.

This study, though relatively small in scope, suggests even a small number of indoor air VOCs can increase certain health risks to unacceptable levels. This is consistent with previous research examining this particular issue^34^. While managing indoor, residential air is problematic from a regulatory perspective, this type of work could, at a minimum, help inform safer product development, product use, and chemical use. This has been common practice in the European Union for many years now and has demonstrated the utility and validity of such an approach. Exposures to indoor air VOCs are often considered voluntary. While it is certainly debatable, exposure to indoor air VOCs is somewhat more controllable at an individual level, as opposed to most outdoor air VOCs where myriad sources contribute^21,26–28^. As mentioned previously, exposures, certainly chronic exposures, to air pollutants are largely a function of indoor air environments, including residences and places of business. To better address actual human health outcomes, health risks, environmental health education, and actionable solutions, exposure determinations and health risk modeling should focus more effort on the indoor air environment.

## Data Availability

All data can be made available by the corresponding author and study principal investigator upon reasonable request.

## Abbreviations

BRFSS: Behavioral risk factor surveillance system
BTEX: Benzene, toluene, ethylbenzene, mixed xylenes
CI: Confidence interval
CR: Cancer risk
GC/MS: Gas chromatography/mass spectrometry
HQ: Hazard quotient
IUR: Inhalation unit risk
K–S: Kolmogorov–Smirnov
MDL: Method detection limit
OVM: Organic vapor monitor
PM_2.5_: Particulate matter with an aerodynamic diameter of 2.5 µm
RfC: Reference concentration
RIOPA: Relationships of indoor, outdoor, and personal air
US: United States
USCDC: United States Centers for Disease Control and Prevention
USEPA: United States Environmental Protection Agency
UTSPH: University of Texas School of Public Health
VOC: Volatile organic compound
WIC: Women, infants, and children
